# It's a numbers game—density-dependent MMP activity mediates cancer cell migration

**DOI:** 10.18632/oncotarget.26115

**Published:** 2018-09-21

**Authors:** Jude M. Phillip, Nahuel Zamponi, Madonna P. Phillip

**Affiliations:** Department of Medicine, Division of Hematology and Oncology, Weill Cornell Medicine, New York, New York, USA

**Keywords:** matrix metalloproteinases, 3D cell migration, cell density, microenvironment, cancer

Metastasis is the leading cause of deaths among cancer patients, particularly within solid tumors. This process involves tumor cells leaving the primary site of disease in response to chemical and mechanical cues, and thereby disseminating to surrounding tissues and organs^1^. Despite its decisive role in disease progression, therapeutic approaches that target metastasis have been elusive. For decades, the primary focus of cancer treatment has been the eradication of cancer cells within the primary tumor. This approach, typically done through the use of chemotherapy and/or radiotherapy approaches induces massive cell killing of both malignant and stromal cells. While beneficial in many instances, the early and/or late dissemination of cancer cells often lead to the recurrence of disease. This observation of directed cellular dissemination away from the primary site, have led researchers and clinicians to evaluate methods to inhibit and slow this dissemination, thereby targeting the metastatic process itself.

In a recent publication, Jayatilaka et al, postulated a novel strategy to target the metastatic process by exploiting key signaling processes involving interleukin 6 (IL6) and 8 (IL8)^2^. They found that as cancer cells grow and divide, the cellular crowding eventually leads to an increase in the spontaneous migration of cells in 3D cultures. Further, they identified that by targeting IL6 and IL8 signaling with Tocilizumab (T) and Reparixin (R) respectively, resulted in a marked decrease in metastatic burden found in other organs using *in vivo* mouse models. Although there were no significant changes in the growth dynamics and sizes of the tumors per se, there was a drastic decrease in the amount of successfully disseminated cells. In this issue of Oncotarget, Jayatilaka et al. builds on their previous work, here discussing the influence of cell density in modulating the expression of matrix metalloproteinases (MMPs), and consequently the migration of cancer cells^3^. They demonstrate using a combination *in vitro* 3D experiments, together with *in vivo* cell line and PDX models, that the modulation of MMP expression arises in part through paracrine IL6/IL8 signaling via the JAK2/STAT3 pathway.

Cells reside in physiologically complex and dynamic microenvironments, both with regards to dimensionality and the physicochemical stimuli that they are exposed to. These external factors often render significant changes on the cellular phenotypes expressed, and the heterogeneity of these phenotypes, for instance in the context normal development and ageing^4^, and disease. As cells grow and divide, there is a constant need to remodel and alter their local surroundings, typically consisting of a dense network of extracellular matrix (ECM) proteins and the stromal cells that secrete them^5^. As such, in order to move and function, cells employ complex mechanisms whereby secreting a class of enzymatic proteins, mainly MMPs, which are able to preferentially degrade various types of fibular and non-fibular ECM proteins (e.g. MMP1 can cleave a subset of collagens, including type I, II, III)^6^. In cancer, where cells exhibit enhanced proliferative capacities and the ability to corrupt stromal components in their favor, this need for remodeling is accentuated. This leads to context-dependent up-regulation of cellular programs for ECM degradation and remodeling; with one such program being through MMP activity. This increase in MMP activity of cancer cells, which was also found to be positively associated with cancer cell motility^7^, was postulated as an attractive target to potentially halt cancer metastasis—the culprit killer of cancer patients. This notion led to the establishment of numerous clinical trials with the goal of targeting aspects of cancer metastasis through MMP inhibition (MMPi). While many of these trials did not perform as expected with regards to enhancing patient prognosis, and resulted in severe side effects in patients^8^, this new understanding of the cell density dependence to MMP activity may provide a previously unappreciated link to aiding therapeutic stratification of patients, i.e. for MMPi.

In the present study, the authors present 3 additional and significant results in our understanding of density-dependence to cancer cell migration. 1) density-dependent regulation of MMP expression mediates cell migration, 2) differential response by cancer cells to MMPi in a cell density dependent manner, and 3) T+R demonstrates favorable responses in animal models with regards to metastatic burden in distal organs, and this signaling indirectly mediates MMP expression. While it remains to be determined what the low density versus high density conditions represent in the setting of patient disease; whether it is based on size of the tumor (small tumors=low density?), or on the degree of stromal infiltration, this study reinforces the claim that cell density, and consequently the signaling of IL6 and IL8 is important, and potentially facilitate patient stratification as a means to target metastasis. Furthermore, as our knowledge of the tumor microenvironment increases, it is becoming more evident that the degree of stromal infiltration and the simultaneous corruption of stromal compartments by cancer cells (of note cancer associated fibroblasts (CAFs)), influences both the progression of disease and the response to treatment. As such, it would be interesting to determine how the presence and abundance of these corrupted stromal compartments influence the density dependence of cancer cell migration and metastasis, and whether the presence of if these CAFs enhances the signaling through IL6 and IL8. In addition, studying co-culture 3D systems of low-density and high-density cancer cells, with varying degrees of stroma and/or immune cells will enhance our understanding of this interaction.
